# Supplementary material for: Cellular and Humoral SARS-CoV-2 Vaccination Responses in 192 Adult Recipients of Allogeneic Hematopoietic Cell Transplantation
Source: Vaccines (Basel). 2022 Oct 23;10(11):1782. doi: 10.3390/vaccines10111782 (PMC9699205; doi:10.3390/vaccines10111782)
Supplement: Supplementary file 1 [file vaccines-10-01782-s001.zip › Supplemental-Text_COVID_alloHCT.pdf]

Supplemental Material

Manuscript: Cellular and humoral SARS-CoV2 vaccination responses in 192 adult recipients of allogeneic hematopoietic cell transplantation

Methods

This retrospective single center study was performed at Freiburg University Medical Center after approval by the local Ethics committee (EK-FR: 21-1590).

Lymphocyte counts

Lymphocyte subsets were determined after full blood staining on an FACS CANTO II™ and analyzed with FACS Diva™ (Becton Dickinson™) at the peri-vaccination period (0-3 months prior to last vaccination) and at one year alfter allo-HCT (330-390 days after allo-HCT).

Statistical Methods

Data were analyzed using R-Studio™ version 1.4.1717, Apache OpenOffice™ version 4.1.7. and Inkscape™ version 1.1.1. For continuous variables normality was assessed visually. Group comparisons of data with a Gaussian distribution were performed using a two sided t test, non normally distributed data was tested using a Wilcoxon rank-sum test. For categorical variables a chi-squared test was used to assess differences. The associations between various parameters and the humoral and cellular response was analyzed using logistic regression models. The models were determined by including relevant factors based on univariate analysis and performing automated stepwise selection of independent variables using a combination of backwards elimination and forward selection.

Supplemental Figure S1: Lymphocyte subpopulations stratified according to vaccination response and timepoint

Box plots display lymphocyte subpopulations up to three months prior to vaccination (peri-vaccination period n = 78 for humoral, n = 49 for cellular response assessment, left column) in both humoral and cellular responders and non-responders. The right column displays lymphocyte counts at one year after allo-HCT in patients vaccinated more than one year after allo-HCT (right column, n = 109 for humoral, n = 58 for cellular response assessment). A) CD19+ B cell, B) CD4+ T cell, C) CD8+ T cell, D) NK cell counts. *, $p < 0.05$

Supplemental Table S1: Multivariate analysis, humoral response failure

Supplemental Table S2: Multivariate analysis, cellular response failure

For the regression model patients vaccinated with only vector based protocols and patients receiving the heterologous protocol were grouped due to the low number of patients in each group. The latter showed a significantly smaller likelihood for cellular response failure when compared to only mRNA based vaccination (OR for non-response compared to mRNA 0.12 , p = 0.01). cGvHD in our univariate analysis did not seem to influence cellular response (univariate 30 % of responders vs 38 % of non-responders with moderate or severe cGvHD, p = 0.50). In our bilinear logistic regression model moderate or severe cGvHD increased the likelihood of cellular response failure upon vaccination (OR for non-response 2.52, p = 0.065).
