# Supplementary figures and images for: Cellular and Humoral SARS-CoV-2 Vaccination Responses in 192 Adult Recipients of Allogeneic Hematopoietic Cell Transplantation
Source: Vaccines (Basel). 2022 Oct 23;10(11):1782. doi: 10.3390/vaccines10111782 (PMC9699205; doi:10.3390/vaccines10111782)

**A B cells**

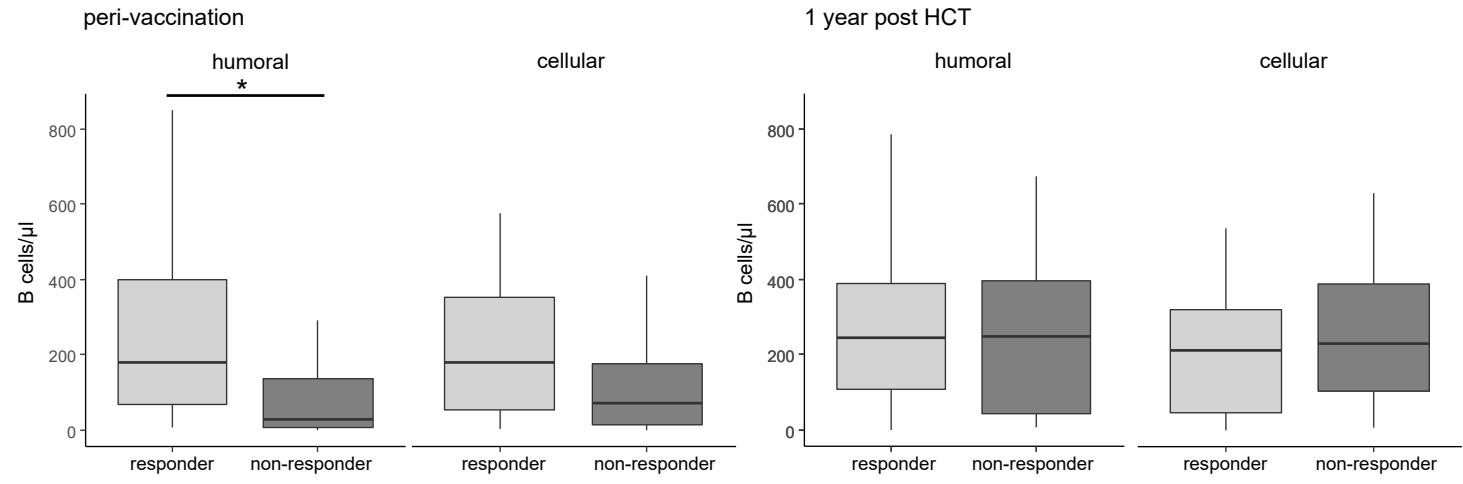

**B CD4<sup>+</sup> T cells**

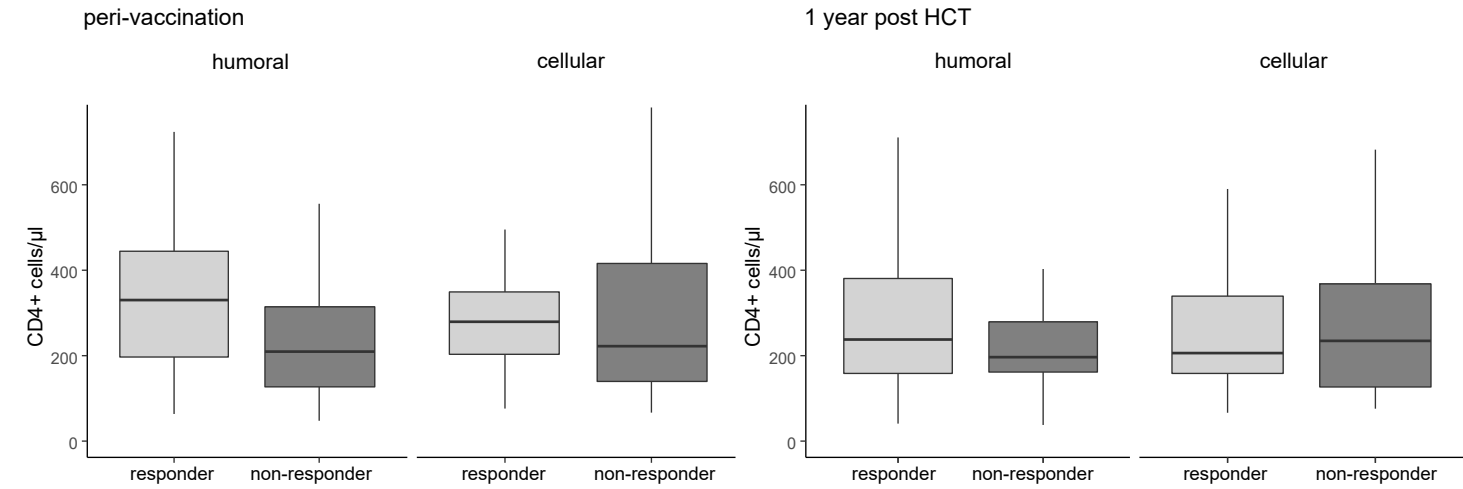

**C CD8<sup>+</sup> T cells**

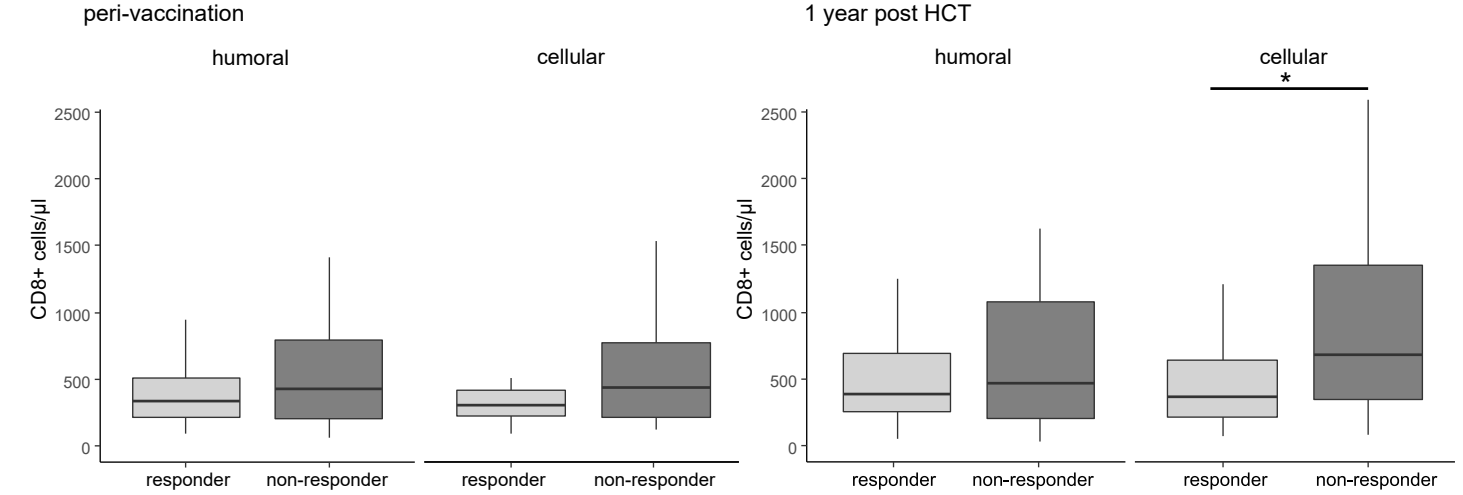

**D NK cells**

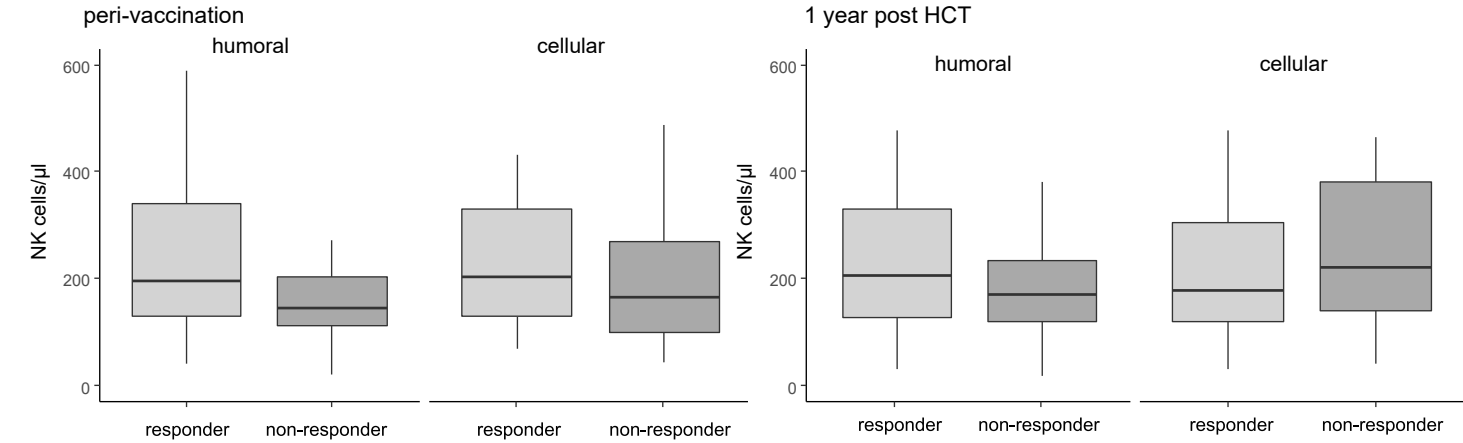

Supplement: Supplementary file 1 [file vaccines-10-01782-s001.zip › Supplemental_Fig_S1.pdf]
